# Pine Beams Retrofitted with FRP and Poplar Planks: Mechanical Behavior

**DOI:** 10.3390/ma12193081

**Published:** 2019-09-21

**Authors:** Francisco J. Rescalvo, Chihab Abarkane, Elisabet Suárez, Ignacio Valverde-Palacios, Antolino Gallego

**Affiliations:** Building Engineering School, University of Granada, Campus Fuentenueva s/n., 18071 Granada, Spain; rescalvo@ugr.es (F.J.R.); elisabetsv@ugr.es (E.S.); nachoval@ugr.es (I.V.-P.); antolino@ugr.es (A.G.)

**Keywords:** poplar wood, pine wood beams, composites, FRP, mechanical behavior

## Abstract

The paper presents an experimental analysis of the bending behavior of pine beams (*Pinus Sylvester*) retrofitted with fiber reinforced plastic (FRP) and poplar planks used as external covering. Poplar wood was chosen because of its rapid growth in planted forests, its homogeneity and attributes for sustainable local development, and high CO_2_ absorption rate. Vibration tests were also conducted in order to evaluate the stiffness in a non-destructive way and compare it with that obtained by means of the destructive tests. Three types of reinforcement were compared, namely: basalt fabric (FB), carbon fabric (FC) and carbon laminate (LC). In addition, some pine beams were reinforced only with poplar planks and used as control specimens in order to evaluate the improvement provided by the FRP. It was observed that a strong delamination preceded the final breakage of beam. Moreover, the results indicated that stiffness is provided mainly by the poplar plank and not by the FRP, as was expected.

## 1. Introduction

The most traditional retrofitting systems of wood structures are made with metallic components (basically steel). As a technological alternative, the use of composite materials for reinforcement, in particular, fiber reinforced plastics (FRP), offers numerous advantages such as lower extra weight and easier installation. Their development has been very important at a global level during the last decades. These lightweight materials are capable of withstanding high mechanical stresses, so that structures are very resistant and very light at the same time. In addition, they have properties ensuring exceptional performance, such as corrosion resistance and high strength. Reinforcing wood beams using FRP materials started in the 1960s [1,2,3], demonstrating that installing fiberglass (GFRP) wrapping under the wood element in a U-shape produces a significant increase of its ultimate strength, an increase of ductility (which is of vital importance for the structural safety), and makes the wood element able to endure greater deflections. Improvements of 50% and 20% in strength and stiffness, respectively, were obtained by some authors by means of the use of GFRP [4,5].

Meier’s work [6] is recognized as one of the pioneers in the use of carbon fiber reinforced plastic (CFRP) as reinforcement of wooden structures. Subsequently, Triantafillou and collaborators [7,8] reported relevant results about the reinforcement with CFRP pultruded laminates placed on the bottom side of the timber: (i) Failure pattern of the specimen changes from brittle to ductile; (ii) a 1% of CFRP material of the cross-sectional area produces an increase in resistance of 60%; and (iii) less CFRP than GFRP material is required to achieve the same effectiveness of the reinforcement (i.e., the CFRP is more efficient that the GFRP in terms of mechanical properties of the reinforced element). Fiorelli et al. [9] indicated that, depending on the type (GFRP or CFRP) and amount of material (from 0.4% to 3%), stiffness increases from 15% to 60%. Valluzi et al. [10] used CFRP sheets, and the ultimate strength improved up to 100%. The great importance of the humidity conditions of the wood during the CFRP-wood adhesion process was also reported. Similar studies can be found in [11,12,13]. 

Buell and Saadatmanesh [14] compared four reinforcement layouts, considering both tension and compression zones for location of the FRP. Improvements were about 17–27% in stiffness, 40–53% in flexural strength, and 36–68% in shear strength. They also considered some layouts of reinforcement that wrapped the lateral sides of the wood beam, demonstrating that this solution produces a substantial improvement in the ductility, due to the lateral shear reinforcement. Similar results were obtained by the authors of [8,15,16,17], who proposed the use of sheets of U-shaped CFRP fabric fully wrapping the bottom and lateral faces of the wood specimen. Since that date, many papers have been published (see [18] for a large description of previous results and literature). 

However, despite this boom in the use of FRP as a reinforcement for wooden structures, there are still much reluctance among professionals and building owners regarding its practical implementation, mainly due to three reasons: (i) Its visual impact; (ii) its high price, especially in the case of FRP carbon; and (iii) its lower sustainability and greater environmental impact and ecological footprint, especially when compared to wood; (iv) The relevance of the bond between materials, either FRP–wood or wood–wood [19,20]. As an alternative to reduce visual impact, this work proposes the use of poplar wood planks to cover the FRP. Poplar wood has good properties that fit perfectly with sustainable construction values [21]: (i) It can be obtained from cultivated forests, reducing pressure on natural forests and promoting sustainable local development; (ii) it is fast growing (between 10–15 years in many areas of the planet), which confers a very high CO_2_ absorption rate when compared with other species; (iii) an adequate management of the plantations produces very good quality wood, free of knots and with much homogeneity, being able to reach values of modulus of elasticity (MOE) even slightly higher than 10,000 MPa; (iv) it has a high resistance, MOR (modulus of rupture), comparable to that of many conifers; and (v) it has a very low density, which is interesting from a structural point of view.

In this context, the present work compares the mechanical behavior of three FRP reinforcement configurations (basalt fabric, carbon fabric and carbon laminate), in which epoxy resin has been used as adhesive for the FRP-wood joint typically used in the case of FRP. In all cases, a poplar plank is also used to cover the reinforcement. As a reinforcement alternative, the poplar plank layout without any FRP is proposed and analyzed using a polyurethane resin as is typically used for the wood–wood joint of the laminated beams. The experimental results analyze the resistance and the stiffness improvements of the four configurations. Also, a theoretical analysis based in the parallel axis theorem is presented.

## 2. Materials and Methods

### 2.1. Pine Beams

Twelve *Pinus sylvestris* wooden beams provided by the company Maderas Pinosoria S.L. (Casarejos, Soria, Spain) with a cross section of 90 × 60 mm^2^ and a length of 1240 mm were randomly selected and used as base beams. For its characterization, the density (*ρ_p_*) and the dynamic MOE (modulus of elasticity) *MOE_din_*_,*p*_, were obtained by means of a longitudinal free vibration test along the grain direction. All the wood characterization tests were performed at a 12% moisture content. Values are shown in Table 1.

### 2.2. Poplar Planks

The poplar wood used for the planks was extracted from a crop of the cultivar I-214 (*Popululs x euramericana* (*Dode*) *Guinier “I-214”*) located at Yunquera de Henares (Guadalajara, Spain). In particular, poplar planks with a cross section of 30 × 60 mm^2^ and a length of 960 mm were extracted from three different trees. With the remaining wood from each of these trees, four-point bending tests were carried out and measurements of the density at the moisture content of 12% were obtained, thus obtaining the static modulus of elasticity (*MOE_st_*_,*po*_), the modulus of resistance (*MOR_po_*), and the *ρ_po_*, as is shown in Table 1.

### 2.3. Reinforced Beams

All twelve pine beams were retrofitted with four different layouts (three beams of each particular configuration). Three of them were reinforced only with poplar planks. For the rest, three types of FRP reinforcements were used: basalt fabric (FB), carbon fabric (FC) and carbon laminate (LC). Details of the dimensions, types of reinforcements, and resins used for each configuration are shown in Table 2. For the pine/FRP/poplar layouts with fabric FRPs (FB and FC), bi-component epoxy resin supplied by MAPEI SPAIN S.A.^®^ (Barcelona, Spain) was used. Bi-component epoxy resin supplied by DRIZORO S.A.U.^®^ (Madrid, Spain) was used for the case of pine/FRP/poplar layout with carbon laminate (LC), while mono-component polyurethane resin PUR-20 supplied by Bakar^®^ (Vizcaya, Spain) was used for the case of the pine/poplar layout. The workability time for the three resins is 40, 60 and 60 min at 23 °C, respectively, while the Brookfiel viscosity is 7000 (20 °C), 300 (23 °C) and 8200 (23 °C) mPa·s, respectively.

Moreover, in the case of the pine/poplar beams, since both materials have similar elastic properties, the combined modulus of elasticity *MOE_st_*_,*c*_ can be theoretically obtained using Equation (1) (the parallel axis theorem or Huygens-Steiner theorem [22]):

(1) $$MOE_{st,c}=\;\overset{N}{\underset{i=1}{\sum }}\frac{E_{i}\cdot I_{i}+A_{i}\cdot E_{i}\cdot y_{i}^{2}}{I_{c}}$$

where $E_{i}$ is the elastic modulus, $I_{i}$ is the second moment of inertia in respect to its own axis, $A_{i}$ is the cross-section area, $y_{i}$ is the distance from the combined neutral axis to the neutral axis of each element for each particular wood species, *i*. $I_{c}$ is the combined second moment of inertia.

In order to ensure an optimal moisture content, both wood species were conditioned at 20 ± 1 °C and 65 ± 5% relative humidity in a climatic chamber. The reinforcement process followed the steps described in [18] with a 12% mean moisture content of the beams. In this case, the curing time of the most unfavorable resin, i.e., the epoxy resin (7–10 days), was taken for all beams. After the reinforcement process, the beams were sanded in order to eliminate any cross-section imperfections that may have been generated during the application of the poplar planks. Once the elaboration process was finished, the beams were relocated into the climatic chamber in order to ensure an adequate moisture content during the tests.

### 2.4. Vibration Test

All the specimens were supported by their geometric center and subjected to free vibration tests conducted along the longitudinal direction (grain direction). A Fakopp SD-02 accelerometer (Fakopp Entreprise Bt., Agfalva, Hungary) was placed at one end of the beam, hitting a hammer on the opposite end. The response signal was recorded with a Picoscope^®^ 4424 oscilloscope (Pico Technology, Wyboston, UK) with 80 MS/s and the fundamental resonance frequency of each beam was obtained, *f*_1_. With this frequency, and assuming the yielding and propagation of one-dimensional volume waves, the dynamic modulus of elasticity of the beam, *MOE_din_*, can be estimated as [23,24]

(2) $$v=2Lf_{1}$$

(3) $$\;MOE_{din}=\rho v^{2}\;$$

where *v* is the propagation velocity of the elastic wave, *L* is the beam length and *ρ* is the beam density.

### 2.5. Four-Point Bending Test

**Figure 1:**
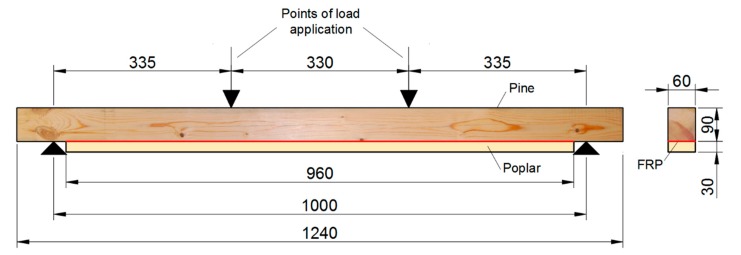
Reinforced beam design and four-point bending test set-up. Distances in mm.

**Figure 2:**
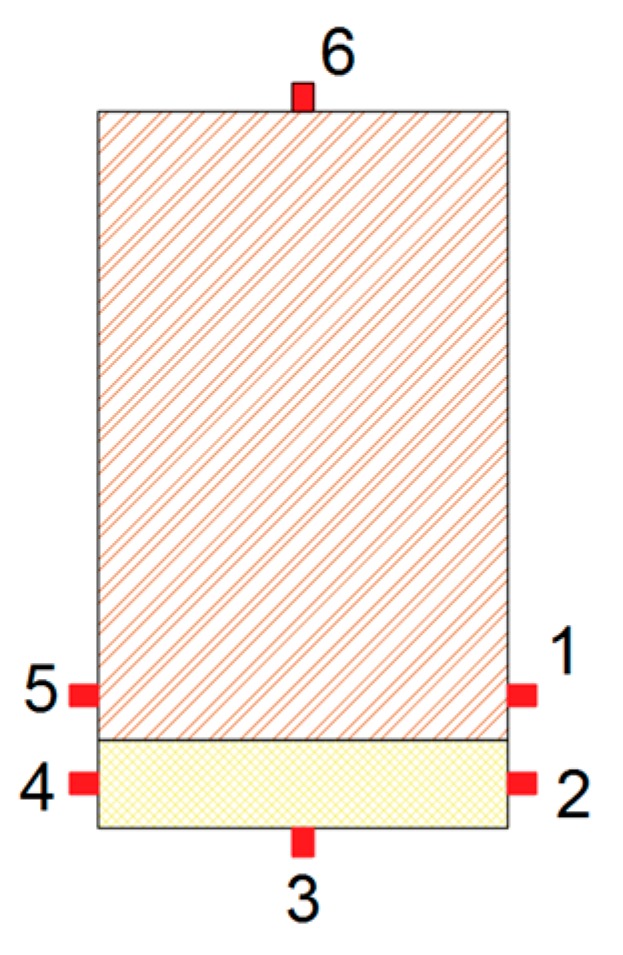
Location of the strain gauges along the mid-cross section of the reinforced beams.

All the specimens were subjected to a four-point bending test, as shown in Figure 1, following the UNE-EN 408: 2011 + A1: 2011 standard [25]. A testing machine model S-110 from CONTROLS S.A (Toledo, Spain) was used, with an electric actuator with a maximum load capacity of 100 kN. In order to measure the maximum tensile strains in the center of the beam and compare the strains of pine and poplar wood, 6 strain gauges were glued on the mid-cross section of each beam, as shown in Figure 2. From the bending tests, the MOR and the static *MOE_st_* were obtained. *MOE_st_* was calculated as the slope of the stress-strain plot between 20% and 50% of the MOR. For this, the strain of the gauge located on the face of maximum tensile stress was used (gauge 3). Gauge 6 measured the strain in the maximum compression area. 

## 3. Results and Discussion

**Figure 3:**
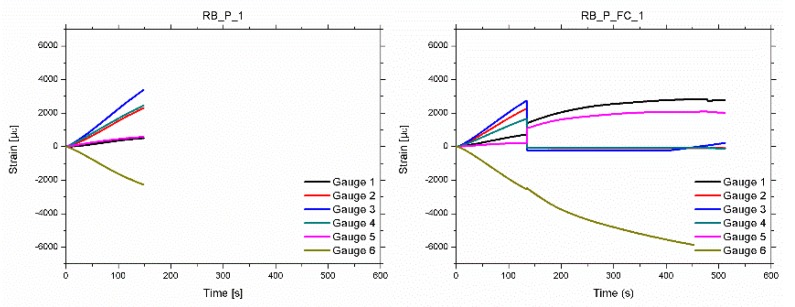
Strain versus time for beams RB_P_1 (failure mode type A) and RB_P_FC_1 (failure mode type B).

The strain measured by the six gauges as a function of time are shown on Figure 3 for two particular specimens (pine/poplar and pine/FRP-FC/poplar beams). In both cases, as expected, the maximum strains were obtained at the upper (tensile) and bottom (compression) external faces. Similarly, due to the symmetrical position, the strain measured by gauges 2 and 4 were similar to each other. This also occurred between gauges 1 and 5. Comparing the strain at the poplar plank and the strain at the adjacent points at the pine beam, greater values were observed at the poplar plank, due to the pine being closer to the neutral line. The mean value of the maximum tensile strain value, at which breakage occurs, was 3444, 3673, 3318 and 3261 με, for the RB_P, RB_P_FB, RB_P_FC and RB_P_LC layouts, respectively.

As shown in Table 1, the poplar plank is stiffer than the pine beam. This causes a significant difference in the strains between the two types of wood, which cannot be absorbed by the resin. This induced the aforementioned delamination. As an example, in Figure 3, it can be observed that for the RB_P_1 beam, this difference in strains is very significant (around 136% at the moment of the final failure). This can be the cause of the final delamination.

**Figure 4:**
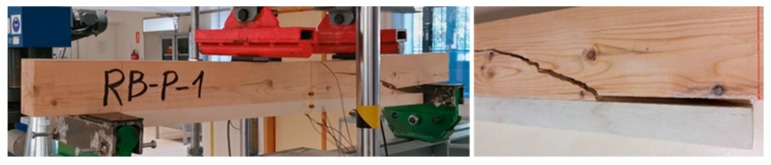
Picture of the final delamination and pine fracture. RB_P_1 specimen. Type A.

**Figure 5:**
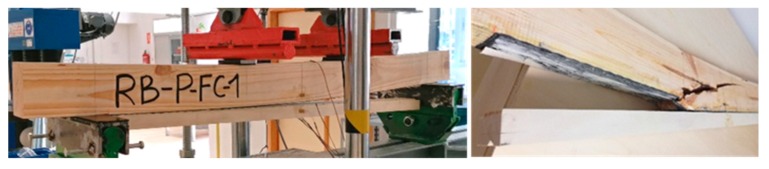
(**Left**) Picture of the delamination during the test at an intermediate load value; (**Right**) pine fracture at the end of the loading. RB_P_FC_1 specimen. Type B.

Two types of mechanical behaviors were observed:

- **Type A**: Beams in which the strains increase permanently until the end of the test. The final failure was caused by a FRP-wood or poplar-pine delamination, followed by a sudden and brittle fracture of the pine beam. Specimen RB_P_1 (Figure 4) is an example of Type A behavior. Moreover, three failure modes were observed in the pine beams after delamination: Type 1: Shear; Type 2: Tensile; Type 3: Shear–tensile mixture.
- **Type B**: Beams in which the strains increase permanently up to a particular intermediate load, in which FRP-wood delamination occurs. At this point, there is a sudden drop in the load and the strains, particularly in the tensile area. At this moment, the strains in the poplar plank become very low, because it is detached. After this drop, a gradual rise of the load occurs until the final brittle fracture of the pine beam, following one of failure modes previously mentioned: 1, 2 or 3. Specimen RP_P_FC_1 (Figure 5) is an example of beams with a Type B behavior.

**Figure 6:**
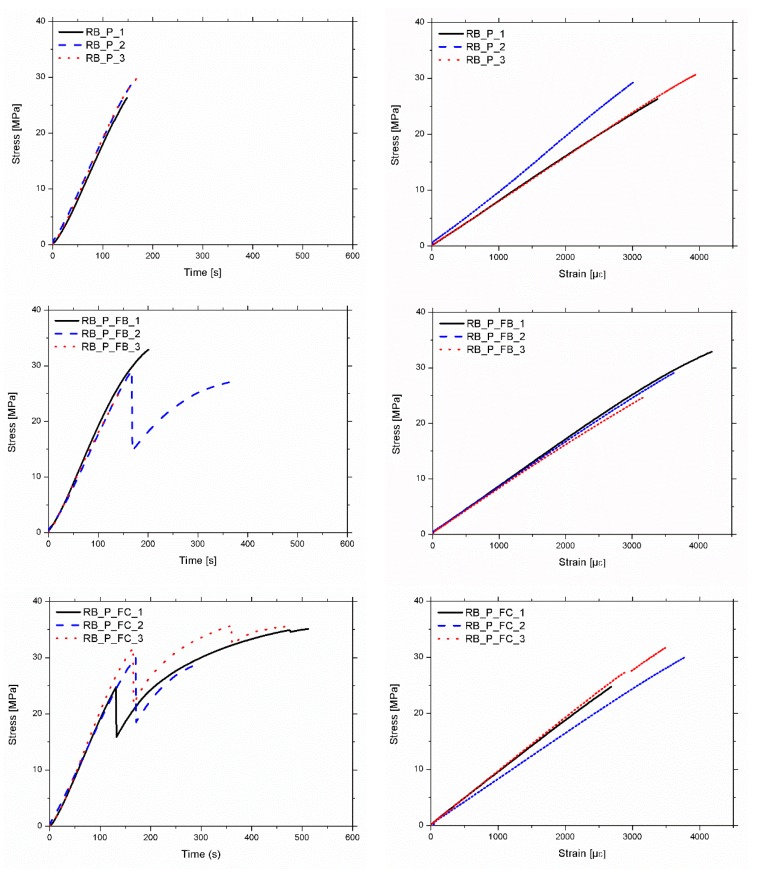

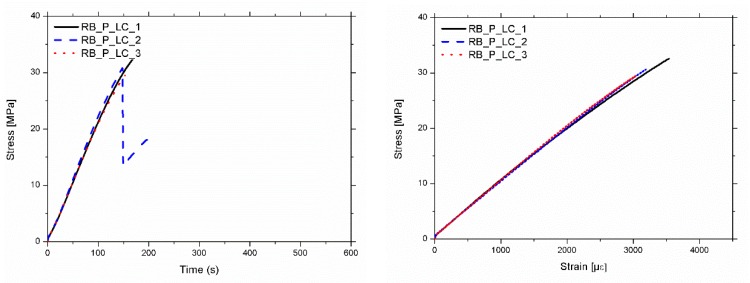
(**Left**) Stress–time plots. (**Right**) Stress–strain plots.

Figure 6 shows the stress-time and stress-strain diagrams for the particular case of gauge 3. Table 3 shows the summary of the mechanical results, including the MOE (static and dynamic) and the MOR. The variation of the dynamic MOE, before and after reinforcement is also shown, in order to compare the influence of the reinforcement. Firstly, it is observed that all the reinforced beams have a lower density than base pine beams (between 7% and 9% less). This is due to the lower density of the poplar wood, despite the mass introduced by the FRP and the resins.

The average MOR of the twelve beams is 30.6 MPa with a low standard deviation, despite the heterogeneity of the base pine wood. It is observed that the average values for the carbon reinforcement layouts (FC and LC) are slightly higher than those without FRP (17% and 8%, respectively) and basalt fiber (FB). This homogeneity of the results between beams, with and without FRP reinforcement, is due to the fact that the main failure is induced by a strong delamination. Keeping these results in mind, and also considering the different distribution of strains between the two pieces of wood (pine and poplar), delamination seems to occur due to the difference in the mechanical behaviour between both species, not due to a lack of FRP/wood bonding.

Comparing the dynamic modulus before (*MOE_din_*_,*p*_) and after reinforcement (*MOE_din_*), an improvement between 14.8% and 20.2% is obtained. However, the increase obtained by using only the poplar plank (18%) is very comparable, or even greater, than that obtained when using FRP. This demonstrates that the improvement is basically provided by the poplar plank, since it has a similar, or even greater MOE, than the base pine beam. This fact is remarkable in pine/poplar beams, since the *MOE_din_*_,*p*_ of pine beams is 6730 MPa on average, compared to the 10,261 MPa of static modulus of poplar planks (*MOE_st_*_,*po*_). It is important to note that dynamic modulus is obtained from a vibration test longitudinally to the wood grain, thus being able to hide the improvement of the FRP. This effect is more evident if the static modulus obtained from the mechanical test (*MOE_st_*) is compared with the theoretical *MOE_st_*_,*c*_ calculated for the pine/poplar beams (RB_P specimens). In this case, the mean variation between the theoretical and the experimental elastic MOE is very low (−4%). It is also remarkable that the pine/FRP/poplar specimens provide very similar values to the theoretical calculation of the MOE without taking the FRP into consideration (3%, 1%, 1% of variation for the FB, FC and LC layouts, respectively). These results seem to indicate that:

- When there is a wooden reinforcement element (as poplar plank) with a considerable thickness and mass compared to that of the FRP (30 mm for the poplar plank versus 0.14 or 1 mm for the FRP), and its MOE is greater or comparable to that of the base beam (around 10,000 MPa for the poplar wood versus 8000–9000 MPa for the pine wood), the contribution of the FRP becomes less relevant.
- As stated before, the failure mode in all configurations is caused by a significant delamination. This makes the MOR values modest and lower than those of the poplar plank, which never breaks. This delamination seems to have been premature and motivated by a lack of adhesion or by an excessive shear stress in the area close to the lower supports.

## 4. Conclusions

A mechanical characterization of pine beams reinforced with FRP and poplar wood planks has been carried out. Three types of pine/FRP/poplar configurations were evaluated (basalt fabric (FB), carbon fabric (FC) and carbon laminate (LC)), which were compared with the pine/poplar one. 

Two failure modes were observed: (i) Type A, in which the load had an increasing tendency until a final brittle failure caused by the FRP–wood or wood–wood delamination; (ii) Type B, in which, after the preliminary delamination, the specimen still retained some load capacity, mainly due to the fibers which remained adhered or partially adhered to the pine base beam. In both cases, FRP-wood or wood-wood delamination was produced at similar MOR values (30.6 MPa), causing high damage to the base beam. This delamination could be associated with the differences in the mechanical behavior of the two wood species and a high stress concentration at the support areas.

Comparing the dynamic elastic modulus, *MOE_din_*_,*p*_ and *MOE_din_* before and after reinforcement, it was observed that the main improvement was due to the poplar plank instead of the FRP. This fact is verified by comparing the theoretical combined modulus, *MOE_st_*_,*c*_, and the static modulus, *MOE_st_* (less than 1% difference). This suggests that the use of a wood element of relevant thickness and mass with an elastic modulus comparable to or greater than the base beam (8000–9000 MPa), makes the contribution of the FRP scarce or almost null. A wider analysis will be conducted in the future, including numerical simulations and a higher number of specimens.

## Figures and Tables

**Table 1:** Mechanical properties of pine beams and poplar planks.

Beam	*MOE_din_*_,*p*_ (MPa)	*ρ_p_* (kg/m^3^)	*MOE_st_*_,*po*_ (MPa) *	*MOR_po_* (MPa) *	*ρ_po_* (kg/m^3^) *
RB_P_1	5616	544	10,261	38	374
RB_P_2	6465	538			
RB_P_3	8108	528			
RB_P_FB_1	7989	485	9441	42	357
RB_P_FB_2	7709	544			
RB_P_FB_3	7239	553			
RB_P_FC_1	8411	523	9441	42	357
RB_P_FC_2	7186	481			
RB_P_FC_3	11,000	591			
RB_P_LC_1	11,773	610	10,142	48	400
RB_P_LC_2	9203	571			
RB_P_LC_3	8775	544			
Mean value	**8290**	**543**	**9821**	**43**	**372**
Standard deviation	**1754**	**38**	**400**	**4**	**18**

* indicates that the values are identical for three beams of the same reinforcement configuration.

**Table 2:** Reinforced beams: Nomenclature, types and main FRP characteristics.

Configuration	Reinforcement Type	FRP	Resin	Consumption (g/m^2^)	FRP Thickness (mm)	FRP Grammage (g/m^2^)
RB_P	Poplar	-	PUR-20		-	-
RB_P_FB	FB/Poplar	Basalt Fabric	MAPEWRAP 21	280	0.14	400
RB_P_FC	FC/Poplar	Carbon Fabric	MAPEWRAP 21	930	0.17	300
RB_P_LC	LC/Poplar	Carbon Laminate	MAXEPOX CS	800	1.4	-

**Table 3:** Mechanical results for the 12 tested specimens.

Beam	MOR (MPa)	*MOE_st_* (MPa)	*MOE_st_*_,*c*_ (MPa)	*MOE_din_* (MPa)	Variation of *MOE_din_* Respect to *MOE_din_*_,*p*_ (%)	Variation of Mean *MOE_st_* Respect to Mean *MOE_st_*_,*c*_ (%)	*ρ* (kg/m^3^)	Variation of Mean *ρ* Respect to Mean *ρ_p_* (%)	Type of Failure
RB_P_1	26.3	8120	7648	6972	24.1		507		A-1
RB_P_2	29.4	9590	8126	7948	22.9		494		A-3
RB_P_3	30.6	7940	8050	8891	9.7		489		A-1
Mean value	**28.8**	**8550**	**8275**	**7937**	**18.0**	**−4**	**497**	**−7.5**	
Standard deviation	**2.2**	**905**	**713**	**960**	**8**	**15**	**9**	**0.7**	
RB_P_FB_1	32.9	8450	8624	9023	12.9		454		A-1
RB_P_FB_2	29.1	8160	8467	8993	16.6		495		B-2
RB_P_FB_3	24.9	8000	8202	8321	14.9		504		A-1
Mean value	**29.0**	**8203**	**8431**	**8779**	**14.8**	**3**	**485**	**−8.1**	
Standard deviation	**4.0**	**228**	**213**	**397**	**1.9**	**1**	**27**	**1.5**	
RB_P_FC_1	35.1	9260	8862	9945	18.2		479		B-3
RB_P_FC_2	30.1	8180	8173	8994	25.1		452		B-3
RB_P_FC_3	35.7	9590	10,318	12,063	9.7		532		B-3
Mean value	**33.6**	**9010**	**9117**	**10,334**	**16.6**	**1**	**488**	**−8.1**	
Standard deviation	**2.8**	**709**	**1095**	**1539**	**7.7**	**6**	**40**	**2.0**	
RB_P_LC_1	32.6	9690	11,059	13,458	14.3		559		A-3
RB_P_LC_2	30.8	9760	9614	11,095	20.6		523		B-1
RB_P_LC_3	29.5	10,009	9373	11,237	28.1		496		A-1
Mean value	**31.0**	**9820**	**10,015**	**11,930**	**20.2**	**1**	**526**	**−8.5**	
Standard deviation	**0.8**	**130**	**912**	**447**	**4.5**	**10**	**17**	**0.3**	
Global mean value	**30.6**	**8896**	**8960**	**9745**	**18.1**	**0**	**499**	**−8.1**	
Standard deviation	**3.2**	**814**	**992**	**1873**	**6.1**	**9**	**30**	**0.4**	
